# Rethinking ‘Innovation’ in Psychiatry with Older and Newer Treatments: From Bench to Benchside

**DOI:** 10.2174/1570159X2111230804161745

**Published:** 2023-09-01

**Authors:** Domenico De Berardis, Giovanni Martinotti

**Affiliations:** 1 Department of Mental Health, ASL 4 Teramo, Italy;; 2 Nursing School, University of L'Aquila, Italy;; 3 Department of Neuroscience, Imaging, Clinical Sciences, University G. d'Annunzio of Chieti-Pescara, Chieti, Italy;; 4 International Centre for Education and Research in Neuropsychiatry (ICERN), Samara State Medical University, Samara, Russia;; 5 Department of Clinical and Pharmaceutical Sciences, University of Hertfordshire, Hatfield, UK;; 6 National Health Service, Department of Mental Health, Contrada Casalena, 64100 Teramo, Italy

In recent years, there have been significant innovations in the field of psychiatry that have revolutionized the way mental illnesses are diagnosed and treated [1]. These advancements have not only improved patient outcomes but have also reduced the stigma surrounding mental health. This mini-special issue is aimed at highlighting some of the key innovations in psychiatry and their impact on the field.

One of the most notable innovations in psychiatry is the development of modern psychopharmacology and somatic treatments, which refers to the use of medication to treat mental disorders [2]. Modern psychopharmacology, the study of psychiatric medications, is a rapidly evolving field. There are several groundbreaking innovations that are changing the landscape of this discipline.

1) Personalized Medicine: Instead of using a one-size-fits-all approach, there's a growing trend toward personalized medicine. This includes genetic or genomic testing to identify specific biomarkers that predict responsiveness to particular psychiatric drugs. It can optimize drug selection and dosages to improve efficacy and reduce side effects.

2) Novel Antidepressants: Ketamine and its metabolite, esketamine (a nasal spray), have been recently approved by the FDA for treatment-resistant depression. They have a quick onset of action (hours to a few days), which is revolutionary compared to traditional antidepressants that take weeks to exert their effects.

3) Psychedelic Therapy: Psychedelic substances like psilocybin mushrooms, MDMA, and LSD are being studied in clinical trials for their potential therapeutic effects on depression, PTSD, and anxiety.

4) Development of Drugs Targeting Neuroinflammation: Research has suggested that inflammation may play a significant role in mental health conditions. This has led to the development of drugs targeting neuroinflammation, representing a new frontier in treating psychiatric disorders.

5) Advances in Transcranial Magnetic Stimulation (TMS): While not a pharmacological treatment, novel developments in neuromodulation techniques like TMS - a noninvasive procedure that uses magnetic fields to stimulate nerve cells in the brain - are changing the way treatment-resistant mental health conditions are managed.

6) Long-Acting Medications: To improve adherence to medication regimens, extended-release formulations and long-acting injectable versions of antipsychotics and antidepressants are being developed.

These innovations are only the beginning of psychopharmacology, and as research progresses, even more, exciting and effective treatments for psychiatric disorders will likely be discovered. In the present mini thematic issue, Mosca *et al.* [3] conducted a systematic review that evaluated the use of ibogaine/noribogaine in the treatment of substance use disorders, and the results demonstrated some efficacy of ibogaine in the treatment of substance use disorders, but the cardiotoxicity and mortality of such drug were worrying and deserving further tolerability studies. Besides, Di Nicola *et al.* evaluated the effect of extended-release trazodone on clinical and functional features in 100 patients with Major Depressive Disorder comorbid with Alcohol Use Disorder. They found that extended-release trazodone reduced depressive symptoms with a remarkable rate of 54.5% concerning remission after six months of treatment. As well analogous improvements were detected in several other outcomes, including anxiety, sleep alterations, and craving. On the other hand, Corkey *et al.* [4] analyzed data provided by the National Records of Scotland concerning individual drug poisoning-related drownings registered from 1996-2020. They found that Scottish drownings associated with drug consumption were increasing, and depressant drugs (such as opioids, benzodiazepines, and alcohol) are often involved. Besides, “designer” benzodiazepines were a main factor in increasing Scottish drug-related poisoning deaths and were partially responsible for growing numbers of associated drownings. Finally, Dell’Osso *et al.* [5] conducted an expert opinion Delphi panel to evaluate the therapeutic appropriateness of cariprazine in the Management of Schizophrenia and found that cariprazine was significantly effective on both acute and maintenance treatment of schizophrenia, and in improving positive, negative, and cognitive symptoms with a very good tolerability profile.

Another important innovation in psychiatry is the rise of neuroimaging techniques [6]. Neuroimaging technologies, such as positron emission tomography (PET) and functional magnetic resonance imaging (fMRI), allow healthcare professionals to visualize the structure and activity of the brain. These techniques have provided valuable insights into the underlying mechanisms of mental illnesses. For example, researchers have used fMRI to identify specific brain regions that are abnormal in individuals with depression or anxiety disorders. This knowledge has not only advanced our understanding of mental disorders but has also opened up possibilities for targeted interventions and treatments.

Furthermore, the advancement of telepsychiatry has revolutionized the delivery of mental healthcare. Telepsychiatry allows patients to receive psychiatric evaluations and treatment remotely *via* video conferencing. This innovation has addressed many barriers to mental healthcare, such as geographical distance, lack of transportation, and limited availability of mental health professionals. Telepsychiatry has made mental healthcare more accessible, particularly in rural areas with a shortage of mental health services. It has also played a crucial role during the COVID-19 pandemic, ensuring continuity of care for individuals who may not be able to visit healthcare facilities in person.

Additionally, the integration of technology into the field of psychiatry has brought about novel assessment and treatment methods [2]. Mobile apps and wearables that track mood, sleep patterns, and activity levels have become valuable tools for monitoring mental health. These technologies enable individuals to be more involved in their mental health management and allow clinicians to gather objective data to inform treatment decisions. Virtual reality (VR) therapy is another example of how technology is being used to treat mental illnesses. VR therapy creates immersive environments that simulate real-life situations, allowing individuals to confront and overcome their fears in a controlled environment.

In conclusion, there have been significant innovations in the field of psychiatry that have revolutionized the diagnosis and treatment of mental illnesses [7]. The development of psychopharmacology, neuroimaging techniques, telepsychiatry, and the integration of technology have all played a crucial role in improving patient outcomes and reducing stigma [6]. As technology continues to advance, it is likely that further innovations will emerge, providing even more effective and accessible mental healthcare solutions.

Overall, it was a great pleasure working with Editor-in-Chief, Professor Ferdinando F. Nicoletti, Sr. Manager Publications, Miss Rabia Moin, and we are honored for the chance to publish this mini thematic issue in the prestigious international journal Current Neuropharmacology. In addition, we would like to acknowledge the contributions of others who edited and processed the manuscripts to obtain the best final quality at the time of publication.
